# School-based relationships and problematic internet use amongst Chinese students

**DOI:** 10.1371/journal.pone.0248600

**Published:** 2021-03-24

**Authors:** Alimila Hayixibayi, Esben Strodl, Wei-Qing Chen, Adrian B. Kelly

**Affiliations:** 1 School of Psychology and Counselling, Queensland University of Technology, Brisbane, Queensland, Australia; 2 Department of Epidemiology, School of Public Health, Sun Yat-sen University, Guangzhou, China; 3 Centre for Inclusive Education, Queensland University of Technology, Brisbane, Queensland, Australia; National Taiwan University of Science and Technology, TAIWAN

## Abstract

The potential for adolescent mental health problems arising from heavy internet use is significant. There is a need to better understand the risk and protective factors related to problematic internet use (PIU) amongst adolescents. This study examined the role of adolescents’ perceptions of school-based relationships as potential contextual antecedents to problematic internet use. Specifically, 6552 adolescent students (55.9% boys, 13.51 ± 2. 93 years) from 22 primary and middle schools in southern China completed questionnaires to measure the degree of adolescent conflict with peers, teachers and other staff, school connectedness, perceived classroom atmosphere and problematic internet use. Self-reported data was collected using a two-level stratified sampling. Multiple regression analyses showed that conflict with peers and teachers was positively associated with higher levels of PIU, while school connectedness and perceived classroom atmosphere were negatively associated with PIU. An interaction effect was found for conflict with peers and grade level, such that the association between conflict with peers and PIU was stronger for secondary students compared to primary school students. The results support the need for school-based interventions for PIU to include a focus on conflict with peers and teachers, and for a focus on the enhancement of school connectedness and classroom atmosphere.

## Introduction

We know that adolescents across industrialized countries report spending a substantial quantity of daily time on the internet. Evaluations of ‘screen time’ indicate that Chinese adolescents spend 7.56 hours/day on viewing screens, American adolescents spend 7.22 hours/day and Australians adolescents spend 6.23 hours/day [1–3]. While the use of the internet by adolescents can be beneficial, the ‘problematic’ use of the internet has also been shown to affect academic performance [4], subjective wellbeing [5], anxiety [6], depressive symptoms [7], sleep disorders [8], reduced leisure activities [9], obesity [10], and suicidality [4].

Although there is no consensus about the definition of problematic internet use, a common definition is that PIU involves a strong urge to use the internet to the extent that it leads to significant social isolation, health issues and academic difficulty [11–13]. Recent research across six Asian countries (China, Hong Kong, Japan, South Korea, Malaysia and the Philippines) found the between 12.5% and 46.0% of 12–18 years old reported PIU [14]. In research using the Adolescent Pathological Internet Use Scale (APIUS) with Chinese adolescents [13], we previously found that the prevalence rate of PIU was 9.0% in Longhua district, Shenzhen [15]. These rates are similar to those found in past research with Chinese adolescents [16, 17]. Given the high prevalence rates of PIU among Chinese adolescents, it is important to identify risk and protective factors for PIU among Chinese adolescents to guide future longitudinal and interventional studies in China and similar cultures.

School-based relationships are likely to be a key domain of potential risk and protective factors for adolescent PIU. Based on social learning principles, adolescents may learn to use the internet to avoid stressful face-to-face interactions with peers, teachers, or other staff in school settings. For example, Italian high school students experiencing interpersonal difficulties report a preference for avoidant coping strategies as well as PIU [18]. Some emerging evidence from China suggests that negative school-based relationships are associated with PIU. For example, poor relationships with peers [16], and teachers, have predicted high levels of PIU in Chinese adolescents [19].

While these studies indicate that negative school-based relationships are risk factors for PIU, relationship quality is a multidimensional construct, and we know little about the dimensions of relationship quality that are related to adolescent PIU [20]. Further research is therefore needed on what aspects or characteristics of school-based relationships are associated with greatest risk of PIU. Given that interpersonal conflict has previously been linked with poor mental health in Chinese adolescents [21], this study aimed to examine the role of school-based interpersonal conflict upon PIU. To date, no study has specifically measured the association between school-based interpersonal conflict and PIU, however there is some emerging evidence that supports such a link. For example, a recent study conducted in China among 2666 middle school students found a significant relationship between experiences with verbal abuse from teachers and peer/online bullying and internet gaming disorder [22]. While indicative, this study was limited to measurement of one form of negative school-based relationship and measured a very specific form of PIU. Hence there is a need to explore whether similar relationships exist between a broader range of conflictual school-based relationships and more general experiences of PIU in Chinese adolescents.

A second reason that school-based relationships may be associated with PIU is derived from attachment theory [23]. This theory emphasizes the role of emotional connectedness and perceptions of safe/secure relationships with significant others as determinants of mental health outcomes [24]. In the school-based empirical literature, different terms have been used to capture emotional connectedness with others, such as school connectedness, perceived school climate, perceived school satisfaction, and school bonding. There is strong evidence that adolescents’ school connectedness is inversely associated with risky behaviors such as sexual risk taking [25], substance use [26], and antisocial behaviors [27]. There is also evidence that elevated school connectedness is positively associated with mental health [28] and a sense of well-being in adulthood [29]. Based on a sample of 2758 middle school students in southern China, Li et al. showed that in adolescents with lower school connectedness, higher levels of PIU were reported [30]. Likewise, classroom environment and climate, for example, perceptions of teacher receptivity, support and inclusiveness, is likely to be protective of emotional and behavioral problems in Chinese adolescents [31]. Very little previous research has tested the association of school connectedness and classroom atmosphere with adolescent PIU.

The focus of this study therefore was to evaluate whether school connectedness, classroom atmosphere as well as peer and teacher conflict are associated with PIU in a large Chinese adolescent sample. We were also interested in examining how these associations varied with age and gender. As adolescents move through puberty, peer networks become increasingly important to the adolescent [27] and adolescents’ sensitivity to conflict with peers increases [32]. In addition, older adolescents may become more vulnerable to anxiety and depressed mood compared with younger adolescents [33]. We therefore anticipated that school connectedness and interpersonal conflict would be more closely linked to PIU for older compared to younger adolescents. In terms of gender, previous findings show that Chinese adolescent females are subject to higher risk of depression associated with higher interpersonal conflict and lower school connectedness compared to Chinese adolescent boys [21]. Similarly, Stavropoulos and colleagues reported that being in a hostile classroom was associated with female’ PIU but not male PIU in adolescents [34]. Given these gender differences, it is likely that the links between both positive and negative school-based relationships and PIU may be stronger among adolescent girls compared to boys. It was hence also decided to also examine the role of gender and grade level moderators of associations between school-based relationships and PIU.

In summary, we aimed to test the associations between school-based conflictual relationships, school connectedness, and classroom atmosphere and PIU, and to examine how these effects varied by grade level and gender. The hypotheses were that:

1. Higher levels of peer-adolescent conflict, teacher-adolescent conflict, and staff-adolescent conflict will be independently associated with higher levels of PIU;
2. Higher levels of school connectedness and classroom atmosphere will be independently associated with lower levels of PIU;
3. Gender and grade level will act as moderators in all the associations above such that the associations will be stronger in females than in males, and stronger in secondary school students than in primary school students.

## Materials and methods

### Study design and participants

This school-based cross-sectional study, with random stratified cluster sampling, was conducted in Longhua district, Shenzhen, China. In phase 1, 22 schools out of a field of 59 schools were randomly selected. In phrase 2, three classes were randomly selected from each grade out of grades 5, 6, 7, 8, 10 and 11 from selected schools. Students from grade 9 and grade 12 were not included as they were facing highly competitive entrance exams to high school and university, respectively. A total of 6638 students were invited in the study and a response rate of 99.2% was received. According to the WHO’s recommended age for adolescent [35], we only included participants aged 10 to 19 years old resulting in 6552 adolescents being included in the final analysis.

### Data collection

Students completed the survey in their classroom with research assistants supervising the survey completion in the absence of the teacher in May 2015. All items on the survey were written in Chinese. Participation was voluntary and responses were confidential without any identity information in order to ensure privacy.

### Ethical statement

This study was approved by the Ethics Committee of the School of Public Health at Sun Yat-sen University, Guangzhou, China (No. 2015–016). Written informed consent was obtained from all the participants before collecting the data, while written informed consent was provided by the student’s parents.

### Measures

#### Problematic Internet Use (PIU)

Adolescents reported on the extent of PIU using the five-item Adolescent Pathological Internet Use Scale [13]. This questionnaire included 38 items with each item rated on a Likert scale (from *1*‘not true at all’ to *5* ‘true all the time’). Higher scores indicated higher PIU. This scale has previously shown to have excellent internal consistency among Chinese adolescents (Cronbach α = 0.97) [15]. It also reported a good convergent validity compared with other widely used scales (8-item Young’s Internet Addition Test versus Chen Internet Addiction Scale = 0.62 versus 0.77) and a good test-retest reliability of 0.86 [36]. The Cronbach alpha in our study was .966.

#### Conflict with peers/teachers/other school staff

School conflictual relationships were rated using a 5-item scale (from *1* ‘never’ to *5* ‘always’), with higher scores indicating higher levels of conflict (e.g. quarrel, fight, emotional or physical punishment/ bullying) with peers (i.e., school friends)/teachers/other staff (non-teaching school employees). These questions were successfully used by a previous study conducted with Chinese adolescents [37]. Conflict with peers was assessed using 3 items: “In the past 12 months, have you ever had a serious quarrel with your fellow students at school?”, “In the past 12 months, have you been involved in physical fighting with your fellow students at school?” and “In the past 12 months, have any of your fellow students emotionally bullied you (humiliated, teased, or threatened you)?” These items have established predictive validity with Chinese students [37] and have successfully been used in our prior research into risk factors for mental health problems in Chinese adolescents [21]. This scale had an adequate internal consistency among Chinese adolescents in this study (Cronbach α = 0.73).

Similarly, conflict with teachers was measured with the following three items: “In the past 12 months, have you ever had a serious quarrel with your teachers at school?”, “In the past 12 months, have you been emotionally punished (such as being scold, threatened, or humiliated) by your teacher in the past 12 months?” and “Have you ever been physically punished (such as being forced to stand for some time, being beaten with fist or other objects, or being kicked) by your teacher in the past 12 months? These questions have been successfully used in previous research into risk factors for mental health problems in Chinese adolescents [21, 37]. This scale also had a good internal consistency among Chinese adolescents in our study (Cronbach α = 0.75).

Conflict with other school staff was assessed with the following questions sourced: “In the past 12 months, have you ever had a serious quarrel with other staff at school?”, “In the past 12 months, have you been emotionally punished (such as being scold, threatened, or humiliated) by other staff at school in the past 12 months?” and “Have you ever been physically punished (such as being forced to stand for some time, being beaten with fist or other objects, or being kicked) by other staff at school in the past 12 months? This scale had an adequate internal consistency in our study (Cronbach α = 0.71)

#### School connectedness

A 5-item scale measuring school connectedness from the National Longitudinal Study of Adolescent Health [38] was translated into Chinese. Adolescents were asked to answer how strongly they agreed with five options regarding school connectedness such as “I feel safe in my school”, “The teachers at this school treat students fairly” (from *0* ‘strongly disagree’ to *4* ‘strongly agree’). The responses were summed to produce a total score, with higher scores indicating greater school connectedness. This scale had good internal reliability (Cronbach α = 0.80 in the present study) and earlier research also shows a good validity [39].

#### Classroom atmosphere

The measure of classroom atmosphere [40] contained 9 items (e.g. “I feel atmosphere of my class is good”, “I feel our head teacher of class is friendly to students” etc.) rated on a 5-point Likert scale (from *0* ‘strongly disagree’ to *4* ‘strongly agree’). The responses were summed up to produce a total score. Higher scores indicated more positive perceived classroom atmosphere. This scale has previously shown excellent internal consistency (Cronbach α = 0.92–0.98) in Chinese samples [40] and also good reliability and validity [41]. The internal consistency in this study was good (α = 0.84).

#### Demographics

The demographic variables in our study included age, gender, academic ranking, grade level (primary, secondary school), family structure (intact, others), maternal and paternal education level (≤ 9th grade, 10-12^th^ grade, and ≥ undergraduate degree).

### Statistical method

The statistical analyses were performed using SPSS 25.0 (Version 25.0. Armonk, NY: IBM Corp, 2017). Of the total sample size (*N* = 6552), the percentage of missing data for all variables were less than 10%. Missing values were imputed using multiple imputations in SPSS and we then used the pooled value for inferential results. Two-tailed Pearson correlation was used to examine the bivariate associations between the independent and dependent variables. To examine whether there were significant differences between different schools, two-level (individuals nested within schools) linear regression showed that only around 4% of variance (ICC < 0.05) was explained at the school level, suggesting that there was low clustering of observations. Hence, multiple linear regression was used to explore the associations between school-based relationships and PIU, and its subscales among adolescents, after controlling for gender, grade level, maternal education level, paternal education level, family structure and academic ranking. The independent variables were transformed into centered values to avoid multicollinearity prior to regressions. We explored potential moderation effects of gender and grade level on PIU by adding interaction terms (e.g. conflicts with peer × gender/grade) to the regression models.

## Results

### Preliminary analyses

Table 1 shows the demographics of the participants in the study. Of the total sample, 55.9% of participants were male adolescents. The mean age was 13.51 years old (*SD* = 2.93) and 43.7% were primary school students (see Table 1). In terms of family demographics, 91.3% were from intact families, and 10.8% of mothers and 16.0% fathers had undergraduate degrees or above. Table 2 shows the mean and standard deviation of the key variable scores and the corresponding age and academic rank of the participants.

**Table 1:** Demographic characteristics.

	Boys		Girls		Total sample	
Demographic variables	*N*	%	*N*	%	*N*	%
Grade						
Primary school	1734	43.7	1121	43.4	2855	43.6
Secondary school	2233	56.3	1464	56.6	3697	56.4
Family structure						
Intact	3593	90.6	2391	92.5	5984	91.3
Others	374	9.4	194	7.5	568	8.7
Maternal education level						
≤ 9th grade	2585	65.2	1637	63.3	4222	64.4
10-12^th^ grade	950	23.9	671	26	1621	24.7
≥ Undergraduate	432	10.9	277	10.7	709	10.8
Paternal education level						
≤ 9th grade	2164	54.6	1320	51.1	3484	53.2
10-12^th^ grade	1186	29.9	835	32.3	2021	30.8
≥ Undergraduate	617	15.6	430	16.6	1047	16.0

**Table 2:** Mean and standard deviation of variables.

	Boys		Girls		Total sample	
Variables	Mean	SD	Mean	SD	Mean	SD
Age (years)	13.67	3.45	13.27	1.82	13.51	2.93
Academic rank	1.97	1.47	1.51	1.34	1.79	1.43
Conflict with peer	5.28	2.14	4.43	1.76	4.94	2.04
Conflict with teacher	4.57	2.08	3.85	1.5	4.29	1.91
Conflict with staff	3.46	1.28	3.23	0.87	3.37	1.14
School connectedness	13.81	4.68	14.02	4.24	13.89	4.51
Perceived classroom atmosphere	24.13	7.4	23.92	7.02	24.05	7.25
PIU	72.15	30.47	63.51	26.32	68.71	29.20

### Bivariate correlations

Independent correlations (split by gender) between the independent variables and PIU are presented in Table 3. For both males and females PIU was significantly correlated with all included variables. Both school connectedness and perceived classroom atmosphere were negatively associated with adolescents PIU, suggesting that they were potential protective factors for PIU. Conflicts with peers, teachers and other staff were positively associated with PIU indicating that these were possible risk factors for PIU.

**Table 3:** Bivariate correlations between school-based relationships and PIU.

Variables	1	2	3	4	5	6
1. Conflict with peer	-	0.414	0.249	-0.231	-0.298	0.230
2. Conflict with teacher	0.402	-	0.432	-0.388	-0.275	0.227
3. Conflict with staff	0.285	0.483	-	-0.232	-0.175	0.155
4. School connectedness	-0.258	-0.437	-0.272	-	0.641	-0.345
5. Perceived classroom atmosphere	-0.315	-0.342	-0.165	0.634	-	-0.212
6. PIU	0.211	0.194	0.113	-0.217	-0.195	-

*p* ≤ 0.01 applies to all values.

The bottom left triangle applies to males and the upper right to females.

### Hypothesis testing

Hypothesis 1 and 2 were tested using stepwise regression, with conflict with peers/teachers/other school staff, school connectedness and classroom atmosphere as the independent variables and PIU as the dependent variable. Step 1 (in Table 4) of the multiple linear regression shows the unique associations of school-based relationships with adolescents PIU after controlling for the covariates (gender, grade, family structure, maternal and paternal education level, academic rank). It indicated that higher conflicts with peers and conflict with teachers were independently related to greater PIU among adolescents (β = 0.162, p < 0.0001; β = 0.079, p< 0.0001, respectively). In addition, higher school connectedness and perceived classroom atmosphere were independently associated to lower PIU (*β* = -0.083, *p* < 0.0001; *β* = -0.040, *p* < 0.05, respectively).

**Table 4:** Multiple linear regression analysis of association of school-based relationships on adolescent’s level of PIU.

Variables	Step 1		Step 2	
	β	t	β	t
Gender	-0.094	-7.386***	-0.086	-6.707***
Grade	0.139	10.675***	0.135	10.242***
Family type	0.041	3.355**	0.040	3.258**
Maternal education level	-0.023	-1.493	-0.021	-1.396
Paternal education level	-0.017	-1.131	-0.017	-1.122
Academic rank	0.064	5.089***	0.061	4.904**
Conflict with peers	0.160	11.297***	0.119	5.303**
Conflict with peers × gender	-	-	0.014	0.084
Conflict with peer × grade	-	-	0.045	2.187*
Conflict with teachers	0.076	4.866***	0.069	2.551*
Conflict with teachers × gender	-	-	0.028	1.527
Conflict with teachers × grade	-	-	-0.01	-0.399
Conflict with staff	0.002	0.176	0.011	0.452
Conflict with staff × gender	-	-	0.019	1.448
Conflict with staff × grade	-	-	-0.019	-0.776
School connectedness	-0.080	-4.731***	-0.111	-3.646**
School connectedness × gender	-	-	-0.003	-0.123
School connectedness × grade	-	-	0.038	1.409
Classroom atmosphere	-0.040	-2.466*	-0.072	-2.568*
Classroom atmosphere × gender	-	-	0.012	0.591
Classroom atmosphere × grade	-	-	0.035	1.434

*p* values were calculated using multiple linear regression model.

* *p* < 0.05;

** *p* < 0.01;

*** *p* < 0.001

Step 2 of the multiple linear regression included the interaction items (see Table 4). This analysis indicated that even after adjusting for the interaction terms, more conflicts with peers and more conflict with teachers were still independently associated with greater PIU among adolescents (*β* = 0.117, *p* < 0.0001; *β* = 0.075, *p* < 0.001). Higher school connectedness and perceived classroom atmosphere also continued to be independently associated with lower PIU (*β* = -0.104, *p* < 0.001; *β* = -0.080, *p* < 0.001).

Hypothesis 3 was tested using stepwise regression with interaction terms for conflict with peers/teachers/other school staff/school connectedness/classroom atmosphere × gender/grade and PIU as independent variable. A significant positive interaction effect was found between conflict with peers and grade upon the level of PIU (*β* = 0.043, *p* < 0.05), suggesting the effect of conflict with peers on adolescent PIU was statistically stronger in older students than younger students.

A simple slope test (see S1 Fig) revealed that the relation between conflict with peers and PIU was stronger among secondary school students than among primary school students. There were no significant interaction effects between any school-based relationships and gender that affected PIU.

## Discussion

The present study examined the associations between school-based relationships and PIU in a large sample of adolescents in China. The hypothesis of higher adolescent-peer conflict and adolescent-teacher conflict being associated with higher PIU was confirmed. That is, higher levels of adolescent-peer conflict and higher levels of adolescent-teacher conflict were associate with higher levels PIU. The hypothesis of higher school connectedness and classroom atmosphere being associated with lower PIU was also confirmed. Finally, the hypothesis that gender and grade level would moderate these relationships was partly confirmed. That is, the associations between conflict with peers and PIU was stronger in older adolescents compared with younger adolescents. However, there was no evidence of gender or grade level moderating the other associations identified.

Previous studies have looked at the associations between both negative peer-adolescent and teacher-adolescent relationships and PIU [16, 19]. However, prior studies used nonspecific definitions of peer and teacher relationship quality, leaving it unclear as to what aspects of relationship quality might be a potential prevention or intervention target. The present study highlights the role of conflictual relationships with peers and teachers as potential contextual factors for PIU. Given that Chinese adolescents spend a large part of their time in school, and academic performance is a major concern for them, it is understandable that school-based conflict with peers and teachers may be potent unique risk factors for PIU. Further, given that Chinese teachers are held in high esteem as part of Chinese Confucian culture [42], the negative impact for students who experience conflict with their teachers may be marked relative to other cultures. Future studies comparing the relative effects for Chinese and Western cultures are needed.

Our finding that both higher school connectedness and classroom atmosphere were related to lower PIU in Chinese adolescents is also novel. This finding is important in that it highlights that the perceived positive relationships within a classroom may add independent protective value to PIU in addition to broader school connectedness. As such both variables are important to consider when examining the associations between positive school-based relationships and PIU. However, this relationship needs to be further explored in other cultures. For example, a study by Jia et al. found that Chinese student’s perceptions of school climate were higher than their American counterparts. Given that there may be cultural differences in perceived school connectedness, our findings need to be replicated in other cultures [43].

Finally, to the best of our knowledge, no previous study has investigated the moderating effect of gender and grade level upon the associations between school-based relationships and PIU amongst adolescents. Contrary to Hypothesis 3, gender did not moderate any of the associations between the school-based relationships and PIU. However, we did find that grade moderated the association between conflict with peers and PIU, with this association being stronger in older students than younger students. One possible reason for the grade moderation effect in the present study may be that peer relationships carry more substantive meaning as they grow older [44], and conflictual peer relationships may therefore engender coping responses like PIU in older students, which in turn may serve to reduce the possibility of solving interpersonal conflict. Therefore, further research is needed to examine the reasons for this moderation effect between conflictual relationships with peers and PIU among Chinese adolescents.

One possible theoretical mechanism that may help explain our results is the use of avoidance as a coping strategy by adolescents. As shown in a cross-sectional study among Italian students, those who had interpersonal difficulties reported more avoidant coping, and in turn experienced more PIU [18]. In addition, lonely adolescents tend to prefer online communication and are more likely to use the internet as a coping strategy for stressful relationships leading to more PIU [45]. Similarly, recent research has shown that negative teacher-student relationships have an indirect effect on problematic mobile phone use through loneliness and motivation to escape negative emotions [46]. Conversely, it is likely that PIU may also have detrimental effects upon interpersonal relationships. For example, there is evidence that PIU can aggravate conflict between family members [47]. These authors suggest that a potential mechanism for such conflict may be that adolescent’s excessive internet use can lead to less face to face interaction with family, and less time studying, which then leads to increased anger among parents. These relationships are likely to be bidirectional as the increase in parental anger may result in more PIU as avoidance coping by the adolescent to escape dealing with distressed parents. Such bidirectional and cyclical school-based relationships need to be further examined in future studies.

There are a number of practical implications from our findings. Teachers, policy makers, psychologists, and public health workers should be cognisant of the interpersonal conflictual relationships with peers and teachers being potential risk factors for adolescent PIU, while positive and supportive classroom and school-based relations appear to be potential protective factors for adolescent PIU. This might involve training both adolescents and teachers in conflict resolution skills, as well as in prosocial interpersonal skills. In addition, teachers could be trained in creating a safe and inclusive classroom environment aligned with school’s values. While, there is some emerging evidence that family based therapy, aimed at improving relationships between adolescents and parents, may help reduce PIU in teenagers [48], there is a need to examine improving school relationships, to reduce PIU. Previous school-based programs have focused on either improving psychosocial competencies, e.g. problem solving, emotional regulation skills, or reducing other health behaviours, e.g. alcohol use, physical activity to reduce PIU [49].

Key areas for further research include investigating the relative role of family-based relationships as independent risk and protective factors for adolescent PIU in addition to school-based relationships. Second, there is a need to clarify the psychological mechanisms linking school-based relationships with PIU. For example, a future study could also include measures of loneliness, depression or anxiety, and avoidant coping strategies in order to examine the degree to which these variables mediated the associations identified in this study. Finally, future research should further explore other potential moderators in addition to gender and grade. For example, a Korean study found that higher school performance was positively associated with longer internet use for study compared to for general use. It suggested that the purpose of internet use could be potential moderating effect between the associations in this study.

This study has many strengths such as it is based on a large sample size and has a high response rate (99.2%) which results in limited bias associated with pathways to participation [50]. Moreover, the tested relationships between variables were controlled for using covariates that have previously been shown to be strong predictors of PIU, such as gender, grade family structure, parental education, and academic ranking [51]. Also, the study explored a diverse range of school-based relationships (positive and negative relationships) and PIU among Chinese adolescents. However, the study also had limitations that are important to be known. First, given the cross-sectional design of the study, conclusions about causal relationships are not possible. Future longitudinal studies are warranted to clarify the direction of these associations. Secondly, caution needs to be exercised when generalizing these findings to all of China as this survey was only conducted in one district in Shenzhen. Moreover, the self-reported data is purely reflective of the adolescents’ perspective. The strength of the validity of these findings could be improved by using multiple informants (e.g. parents, teachers, and peers) and multiple methods (e.g. interview, and observation). It is also worth noting that the findings of this study were limited to general problematic use of the internet rather than specific categories of problematic use (e.g. gaming addiction, smartphone addiction, social media addiction etc). In addition, the sample only included primary and secondary school students rather than vocational school students who might be more vulnerable to PIU [52].

## Conclusion

This study revealed that conflict with peers and teachers were positively associated with PIU, while school connectedness and perceived classroom atmosphere were negatively associated with PIU in Chinese adolescents. The effect of conflict with peers on PIU was stronger in older than younger students. These findings point to the importance of effective conflict management, especially for older students, and the promotion of school connectedness and perceived classroom atmosphere to decrease PIU among Chinese adolescents.

## Supporting information

S1 Fig Conflict with peers and PIU: The moderating role of grade level. (PDF)
