# Supplementary figures and images for: School-based relationships and problematic internet use amongst Chinese students
Source: PLoS One. 2021 Mar 24;16(3):e0248600. doi: 10.1371/journal.pone.0248600 (PMC7990311; doi:10.1371/journal.pone.0248600)

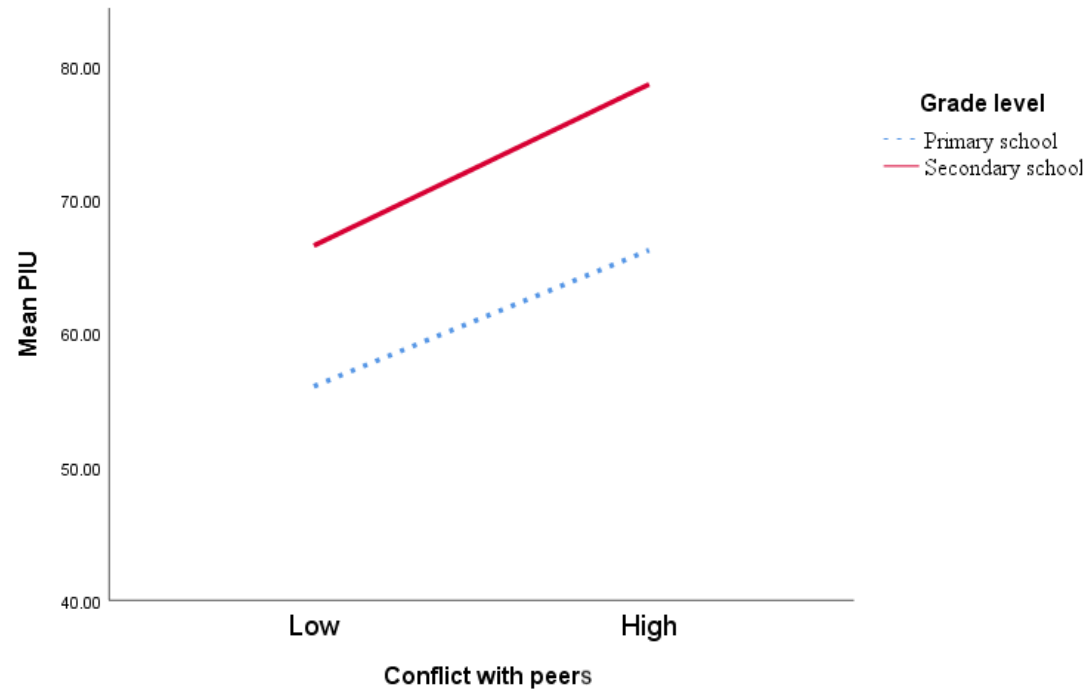

Supplement: S1 Fig — (PDF) [file pone.0248600.s001.pdf]
